# Temporary suppression the sequestrated function of host macrophages for better nanoparticles tumor delivery

**DOI:** 10.1080/10717544.2018.1474965

**Published:** 2018-06-05

**Authors:** Jifu Hao, Te Han, Meixiang Wang, Qiannan Zhuang, Xiaodan Wang, Jianguo Liu, Yongan Wang, Hua Tang

**Affiliations:** a College of Pharmacy, Taishan Medical University, Taian, PR China;; b Institute of Immunology, Taishan Medical University, Taian, PR China

**Keywords:** Macrophages depletion, paclitaxel, PLGA nanoparticles, clodrolip, antitumor effect

## Abstract

Orchestration of nanoparticles to achieve targeting has become the mainstream for efficient delivery of antitumor drugs. However, the low delivery efficiency becomes the biggest barrier for clinical translation of cancer nanomedicines, as most of them are sequestrated in the liver where more macrophages located in are responsible for capture of systemic administrated nanoparticles. In this study, we found that the depletion of the liver macrophages could lead to a superior improvement in the nanoparticles delivery. Firstly, we developed clodronate-containing liposomes (clodrolip) to transiently suppress the phagocytic function of macrophages, the residual macrophages in liver only accounted for less than 1% when the mice were treated with clodrolip in advance. In addition, the pharmacokinetics results of treatment with paclitaxel-poly(lactic-co-glycolic acid) (PTX-PLGA) nanoparticles disclosed that the AUC of PTX in the macrophages depletion group increased 2.11-fold. These results meant that the removal of macrophages would decrease the nanoparticles accumulation in the liver and better the biodistribution and bioavailability of nanoparticles delivery systems. Moreover, treatment of mice with melanoma by the combination of clodrolip and PTX-PLGA nanoparticles resulted in an elevated anti-tumor efficacy, the tumor inhibition ratio was nearly reached to 80%. Furthermore, these combinatorial regimens have demonstrated negligible toxicity in incidence of adverse effects. In conclusion, the encouraging results from this study inspire the generation of a rational strategy to focus on microenvironmental priming for modulation of innate immunity and to improve delivery efficiency of nanoparticles.

## 1. Introduction

Orchestration of nanoparticulates to fulfill tumor targeting has been gradually regarded as a promising approach to address the less specificity of traditional chemotherapeutic agents (Danhier, 2016). Incorporating free therapeutical drugs into nanoparticles can confer them distinctive advantages such as increase site-specific targeting, reduction side effects and improvement therapeutic efficacy (Davis et al., 2008). Now, two conventional strategies, either alteration of physicochemical properties of nanoparticles with optimal particle size and surface charge or modification of nanoparticles with biofunctional ligand molecules, have been formulated and exploited for oriented delivery of nanoparticles to specific organs or even to particular cells, which correspondingly known as passive targeting based on enhanced permeability and retention (EPR) effect and active targeting approach relied on the affinity being strong or not between the ligands on the surface of the nanoparticles and the receptors present on the targeted sites. However whichever type of the targeting strategy we choose, nanoparticulate drug delivery systems have not yet reached to their anticipative functions (Michor et al., 2011; Parodi et al., 2013). Once entering into the circulatory system, systemically injected nanoparticles will encount many barriers on their long route to arrive at the specific destination. As one of the innate biophysically defensive mechanisms of the body, the mononuclear phagocyte system (MPS) answers for identifying the foreign nanoparticulate substances through a process named opsonization; then the alient nanoparticles are captured by resident macrophages in the liver, mainly kupffer cells (Moghimi & Simberg, 2017). Recently a report disclosed that up to 99% of the injected nanoparticles mainly deposited in filtration organs such as the liver and spleen, where a large amount of macrophages resided in; Such low delivery efficiency (less than 1%) of administrated cancer nanomedicines indicated that active agents did not arrive at the desired regions in enough quantities as most of them were sequestered by nontumor cells (Gustafson et al., 2015; Tavares et al., 2017). Indeed, maintenance of nanoparticles circulating in the blood without being eliminated is a cardinal prerequisite for tumor targets in that only those nanoparticles with long circulation properties and without distribution into other non-targeted sites can have the potential opportunities for tumor tropic accumulation in light of EPR effect theory. Hence, sequestration of systemically administrated nanoparticles by MPS became one of the critical roadblocks to compromise nanoparticles precisely locating at the tumor sites (Wilhelm et al., 2016). As a consequence, to function properly, systemic agents must evade clearance by the immune system and then concentrate at the target tissue in adequate quantities (Parodi et al., 2013). A number of methods to fabricate multifunctional nanoparticles had already been exploited to decrease or reduce clearance by the MPS, all of them, pegylation was once proposed as the preferential approach. While more and more studies demonstrated that pegylation of nanoparticles had negligible improvement in evasion MPS clearance (You & Park, 2011). Hence, in order to improve the anti-tumor efficacy of the systemically administrated nanoparticles, we should pay more emphasis on the physiological condition of the body, especially focus on harnessing those macrophages’ functions intended to diminish nanoparticles uptake (Khalid et al., 2016). The progressive studies in microenvironmental priming arouse us to think such a question ‘Does the efficiency of delivery nanoparticles to tumors increase when removal some of the macrophages which sequester them?’

Here we assumed that if the host macrophages were depleted, in particular suppression of the function of those kupffer cells resident in liver, when the nanoparticles travel in the blood stream through the liver, the sequestration of chemotherapeutic nanoparticles of macrophages would be temporarily impaired. As a consequence, the increased drug blood concentrations would enable more and more drugs permeate into the tumor tissue, and consequently promoted their antitumor effects.

Dichloromethylenediphosphonic acid disodium salt (clodronate) was once used for the treatment of bone disease such as osteoporosis. While when it was encapsulated into liposomes, Clodronate-containing liposomes (clodrolip) could be recognized and engulfed by macrophages, and then led to macrophages death via apoptosis (Van & Hendrikx, 2010; Samuelsson et al., 2017). Clodrolip has been generally used as efficient tools to deplete macrophages in the field of immunology (Wolfram et al., 2017).

In this paper, we proposed a combinatorial treatment approach for tumor therapy; at first we used clodrolip in advance to transiently inhibit macrophages’ activity in the MPS, then we chose paclitaxel-poly(lactic-co-glycolic acid) (PTX-PLGA) nanoparticles as antitumor nanocarrier systems to probe whether their antitumor efficacy can be correspondingly improved after removal of the liver macrophages. We have performed the *in vivo* depletion of macrophages experiments and evaluated the biodistributions and antitumor effects of PTX-PLGA nanoparticles in mice after pretreatment with clodrolip. We hope this combinatorial method can serve as a promising approach for cancer chemotherapy and modulate innate immunity to improve delivery efficiency of nanoparticles.

## 2. Materials and methods

### 2.1. Materials and animals

Poly(lactic-co-glycolicacid) (PLGA, 0.64 dL/g) with terminal carboxyl was purchased from Daigang Biotechnology Co., Ltd. (Jinan, China). PTX was supplied by Ciyuan Biotechnology Co., Ltd. (Shanxi, China). Phosphatidylcholine and cholesterol were acquired from Ruixi Biotechnology Co., Ltd. (Shanxi, China). Dichloromethylenediphosphonic acid disodium salt was purchased from Shanghai JONLN Reagent Co., Ltd. Other reagents used in all the experiments were of analytical grade.

C57BL/6J mice were provided by the Taishan Medical University Animal Center (Taian, China). All animal studies were handled according to the Principles of Laboratory Animal Care, and the protocols were approved by the Animal Ethical Committee of Taishan Medical University.

### 2.2. Preparation of clodrolip and selective depletion of macrophages *in vivo*

#### 2.2.1. Fabrication of clodronate-laden liposomes

Application of clodrolip to deplete macrophages is one of the conventional approaches used in the field of immunology. In this experiment, we used thin film and hydration method to tailor clodrolip. Briefly, phosphatidylcholine and cholesterol (molar ratio 4:1) were codissolved in 10 ml of chloroform in a round bottom flask. After complete removal chloroform by rotary evaporation and form a thin film on the interior of the flask, 10 ml of PBS, containing 2.5 g clodronate, was used to hydrate the formed lipid film. Then, the acquired suspension was suffered with a probe ultrasonication to develop clodrolip (Scientz, Ningbo, China). After centrifuged at 3000 rpm at 4 °C for 15 min, the plethora of clodronate was removed and the obtained clodronate liposomes were rinsed with PBS. Subsequently, the clodrolip was resuspended in 4 ml of PBS for further use.

The particle size, polydispersity index (PDI), and morphology of the tailored clodrolip were analyzed by a Zetasizer (Nano ZS90, Malvern Instruments Ltd., UK) and TEM (JEM1200EX, JEOL, Tokyo, Japan), respectively. The content of clodronate was determined using a UV spectrophotometer at 240 nm (UV-8000, METASH Shanghai, China) and then the drug loading (DL) and the encapsulation efficiency (EE) of clodrolip were calculated according to the following calculation equations (van Rooijen & Hendrikx, 2010).

$$\text{DL \%}=\frac{\text{Weight of the drug in clodrolip}}{\text{Weight of the feeding lipid and drug}}\times 100\text{\%}$$

$$\text{EE \%}=\frac{\text{Weight of the feeding drug}-\text{Weight of the free drug}}{\text{Weight of the feeding drug}}\times 100\text{\%}$$

Also, the *in vitro* drug release of clodrolip was performed. The liposomes suspension was introduced into a dialysis bag (MWCO 15 KD) then the dialysis bag was immersed into 100 ml release medium (pH 7.4, PBS) and incubated at 37 °C with constant agitating speed at 100 rpm. Subsequently, 2 ml of samples was withdrawn at different time intervals and substituted with the same volume of fresh medium. The concentration of clodronate in the samples was analyzed at 240 nm by the use of UV spectrophotometer method. Subsequently, the cumulative release curve of clodronate (Q%) was achieved.

#### 2.2.2. Depletion of macrophages *in vivo* by clodrolipo

For evaluation the efficiency of macrophages depletion induced by clodrolip, the mice were injected with clodrolip via tail vein on day 0, then on days 1, 3, 5, 7, 10, and 14 (*n* = 3 for each group), the animals were performed euthanasia by injection of overdosed anesthetic. The liver and spleen were harvested and perfused with the collagenase solutions for preparation of single cells. After complete digestion, macrophages were isolated and stained with F4/80 and CD64 mAb (eBioscience, Thermo Fisher Scientific, USA) to distinguish other cells. Subsequently, staining cells were analyzed by an LSRII flow cytometer (BD Biosciences, San Jose, CA, USA), and the obtained data was treated with Flow Jo software (Tree Star Inc., FlowJo, Ashland, OR, USA).

### 2.3. Preparation and characterization of chemotherapeutic PTX-PLGA nanoparticles

The PLGA nanoparticles were tailored by a traditional O/W emulsification solvent evaporation technique. Briefly, PLGA/drug solutions containing 100 mg of PLGA and 10 mg of PTX were prepared using 4 ml of ethyl acetate. The obtained solutions were then dispersed into 20 ml of a 0.1% of PVA solution and subsequently emulsified using a probe sonicator (Scientz, Ningbo, China) at 400 W for 2 min in an ice bath. The resulting emulsion was stirred for 24 h under a fume hood until the organic solvent (ethyl acetate) volatilized completely.

The particle size, zeta potential, and morphology of the prepared PLGA nanoparticles were characterized by a Zetasizer (Nano ZS90, Malvern Instruments Ltd., UK) and TEM (JEM1200EX, JEOL, Tokyo, Japan), respectively. The content of PTX was determined by HPLC approach (Shimadzu LC-10AT, Tokyo, Japan) and the DL and EE of the nanoparticles were calculated as follows:

$$\text{DL \%}=\frac{\text{Weight of the drug in PLGA nanoparticles}}{\text{Weight of the feeding polymer and drug}}\times 100\text{\%}$$

$$\text{EE \%}=\frac{\text{Weight of the feeding drug}-\text{weight of the free drug}}{\text{Weight of the feeding drug}}\times 100\text{\%}$$

The nanoparticles were dissolved in acetonitrile to determine the total weight of drug in the nanoparticles. The free drug was separated from the nanoparticles using an ultrafiltration method. The HPLC system comprised a LC-10A pump (Shimadzu, Kyoto, Japan) with a UV visible detector. A thermofisher C18 (250 × 4.6 mm) analytical column was used with the mobile phase of acetonitrile and water (45:55). The flow rate was 1.0 ml/min at room temperature.

The *in vitro* release of PTX from PLGA nanoparticles was also performed by dialysis method. Non-encapsulated PTX was removed according to ultracentrifugation method prior to an *in vitro* release test. In order to meet the need of sink condition, PBS (pH 7.4) with 0.1% Tween 80 was selected as release medium. Then, the PTX-PLGA suspension (drug content:2 mg) and PTX suspension with the same content were introduced in a dialysis bag (MWCO 15 KD) and then placed into 100 ml release medium which was incubated at 37 °C under constant stirring speed at 100 rpm. At each presetting time interval, 1 ml aliquots of release medium were withdrawn and supplied with an equal volume of fresh medium. After centrifugation at 13,000 g for 15 min, the supernatant PTX content was determined using HPLC at 227 nm. Then, the cumulative release percentage (Q%) was calculated.

### 2.4. Pharmacokinetics and biodistributions of PTX after injection of PTX-PLGA nanoparticles in mice depleted of macrophages

#### 2.4.1. Pharmacokinetics experiments

To probe whether the plasma concentration of PTX in macrophages-depleted mice might be superior to that in the normal physiological condition, pharmacokinetics of PTX on mice with/without macrophages depletion were carried out. C57BL/6J mice, weighting 18–20 g, were randomly divided into two groups before the experiments. Clodrolip was injected in advance via vein tail 1 day before in order to deplete macrophages in the experimental group; 0.9% of normal saline was injected as the placebo in the control. Then, the same doses of PTX-PLGA nanoparticles (amount to 5.0 mg/kg PTX) were injected on day 0. The whole blood sample (about 0.2 ml) was collected into heparinized tube by sinus jugularis puncture from each mouse in every group at 0.25, 0.5, 1, 3, 6, and 8 h, respectively. Plasm were separated by centrifugation for 10 min at 4000 rpm, and the supernatant was collected and stored at –80 °C for further analysis.

#### 2.4.2. Biodistributions studies

As for tissue distribution studies, the experimental protocol was similar to the aforementioned pharmacokinetics design. At predetermined time, the blood samples and tissues, such as heart, liver, spleen, lung, and kidney, were collected immediately after heart perfusion with PBS, and the plethora of water in the isolated organ was removed with filter paper. The plasma and tissue samples were stored at –80 °C until analysis.

#### 2.4.3. Measurement of plasma and tissue samples of PTX

To determine the contents of PTX in plasma and tissue samples, 100 μl of plasma samples was treated with 300 μl of acetonitrile in a tube to precipitate protein and extract PTX, then vortexed for 3 min and centrifuged for 10 min at 12,000 rpm at 4 °C. After removal of the debris, the supernate was blown to dry with nitrogen gas stream at room temperature. The residue was redissolved with 100 μl of acetonitrile and subsequently performed for HPLC analysis. To measure the tissue distribution of PTX, tissue sample was weighted accurately and homogenized after addition of moderate PBS to obtain 1 mg/ml of homogenate. The rest of the procedure was similar to the treatment of the plasma concentration.

The plasma concentration of PTX versus time profile was analyzed using DAS Version 2.0 (Drug Clinical Research Center of Shanghai University of TCM, Shanghai, China). Various pharmacokinetic parameters were obtained such as the area under the plasma concentration time curve from zero to infinity (AUC_0→∞_) and mean residence time (MRT). All data were expressed as the mean ± SD (standard deviation).

### 2.5. Therapeutic performance of PTX-PLGA nanoparticles in mice after depletion of macrophages

We hypothesized after depletion of macrophages the sequestration of PLGA nanoparticles by MPS would be impaired; in particular those macrophages resided in the liver and spleen, which accounting for the major population of the MPS. Based on this assumption, the suppression of phagocytic functions of macrophages will render systemic nanoparticles long-circulated properties in blood, which providing more opportunities for therapeutic nanoparticles to reach the tumor sites, thereof increase the antitumor efficacy. The *in vivo* tumor growth inhibition efficacy of PTX-PLGA nanoparticles was assessed on 6-week-old female C57BL/6J mice after macrophages depletion. A tumor model was constructed with subcutaneous inoculation of syngeneic B16/F10 melanoma cells. When the tumor volume reached about 100 mm^3^, the mice were sorted randomly into four groups (*n* = 5) and treated with one of the following regimens: (1) saline solutions (the control group); (2) clodrolip injected twice via tail vein at days 0 and 6 without combinatorial administration of PTX-PLGA nanoparticles; (3) PTX-PLGA nanoparticles; and (4) clodrolip pretreatment via tail vein injection at day 0, then received PTX-PLGA nanoparticles with a PTX dose of 0.5 mg/kg on day 1 in a 2-day interval and repeated four times treatments, in order to thoroughly clear macrophages, clodrolip was also injected at day 6 to prevent macrophages repopulation.

The tumor growth inhibition was evaluated by monitoring the tumor volume in the experimental durations. Tumor size was measured by digital calipers and tumor volume was calculated according to the equation: $\text{Volume}\text{=}\frac{\left(\text{tumor length}\right)\text{×}{\left(\text{tumor width}\right)}^{\text{2}}}{\text{2}}$

After the mice were sacrificed at the end of the experiment, the tumors were harvested and weighted, and further the inhibition rate of tumor growth was calculated. Then, tumor growth inhibition rate which was used to assess the therapeutic efficacy against tumor was calculated (Wang et al., 2014).

### 2.6. Adverse effects evaluation of PTX-PLGA in mice

Potential adverse effects in the mice treated with the previous established protocol were also evaluated during the therapeutic experiments. Major organs and the blood samples were collected for histological tissue and/or biochemistry analysis. Tissue samples were embedded in optimal cutting temperature compound and performed cryotomy. Each section was cut into 5 μm, processed for routine hematoxylin and eosin (HE) staining, and then observed under microscope (Olympus BX46, Japan). Blood was collected in tubes containing dipotassium EDTA and kept on ice. For biochemistry analysis, serum was separated from the whole blood by centrifuged at 3000 rpm for 15 min at 4 °C. Biochemical markers to be used for detection the toxicity on the major organs such as lactic dehydrogenase (LDH), aspartate transaminase (AST), alanine transaminase (ALT), total bilirubin (TBIL), uric acid (UA), and creatinine levels (CRE) were analyzed by the specific assay kits (Nanjing Jiancheng Bioengineering Institute, China).

Body weight change was also monitored during the experiment to evaluate the toxicity of different groups. To further evaluate the toxicity, the organs of the mice were harvested at the end of the trial to calculate the organ coefficient, which was calculated using the following formula:

$$\text{Organ coefficient \%}=\frac{\text{weight of the organ}}{\text{body weight}}\times 100\text{\%}$$

## 3. Results

### 3.1. Characterization of clodrolip

**Figure 1.**
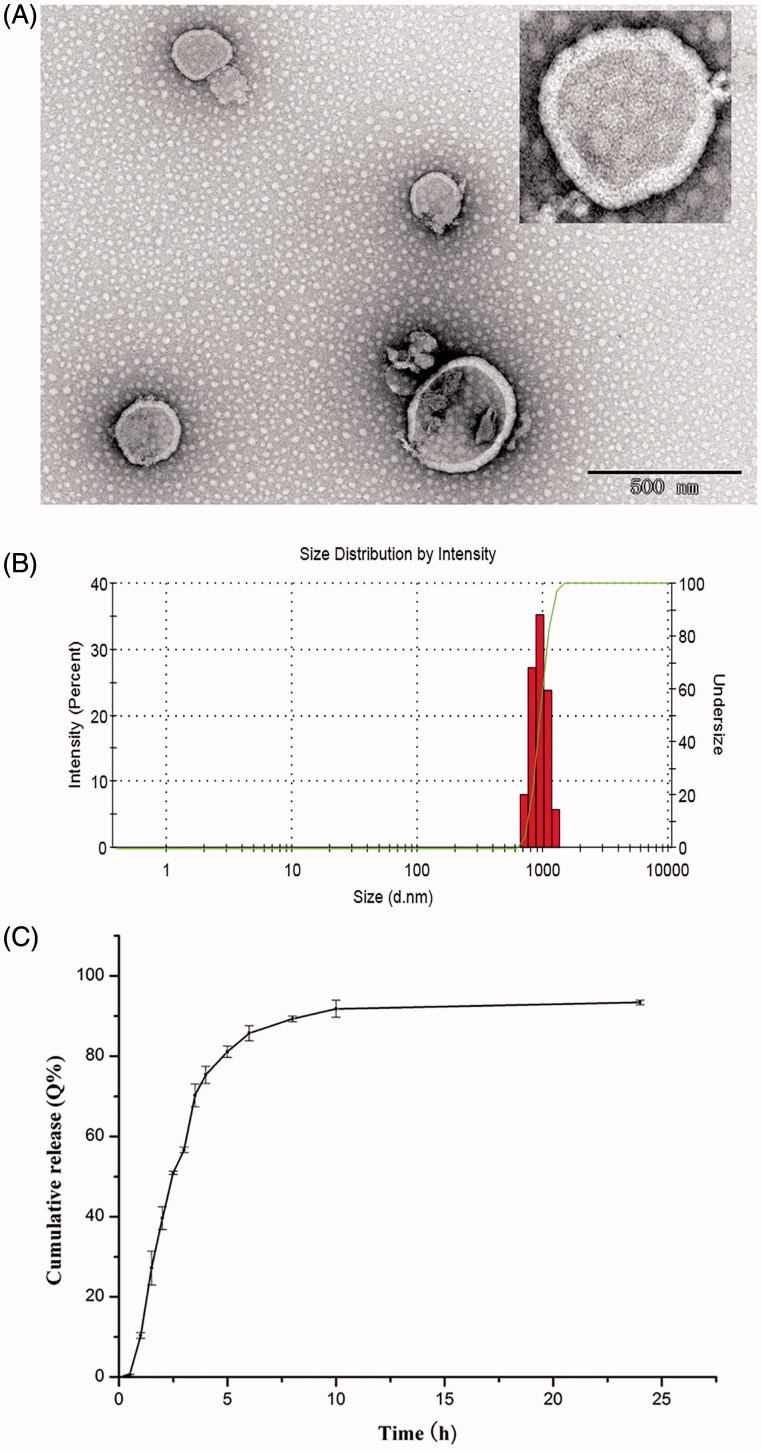
The TEM morphology (A); particle size distribution (B); and *in vitro* drug release profiles (C) of clodrolip.

Only particulate delivery system such as in the form of liposomes can mediate clodronate internalization into macrophages, with the help of liposomes, the fate of clodronate *in vivo* is phagocytosis by macrophages. In this study, clodrolips were fabricated by the conventional thin-film hydration method. The high hydrophilic molecules of clodronate were solved in aqueous solutions which can be encapsulated into the core of liposomes during the hydration process. The surface morphology of clodrolip observed by TEM was shown in Figure 1(A). The liposomes were spherical structure with a smooth surface. The mean diameter of clodrolip was 986.2 ± 37.6 nm (*n* = 3) (Figure 1(B)). The DL and EE of preparation were 1.52 ± 0.084%, 1.6 ± 0.073% (*n* = 3), respectively. The *in vitro* release properties of clodronate were evaluated in PBS (pH 7.4) to mimic the physiological condition. As shown in Figure 1(C), clodrolip presented a constantly sustained release behavior in that the cumulative release of clodronate was nearly reached to 90% within 8 h.

### 3.2. Effective depletion of macrophages by pre-administration of clodrolip

**Figure 2.**
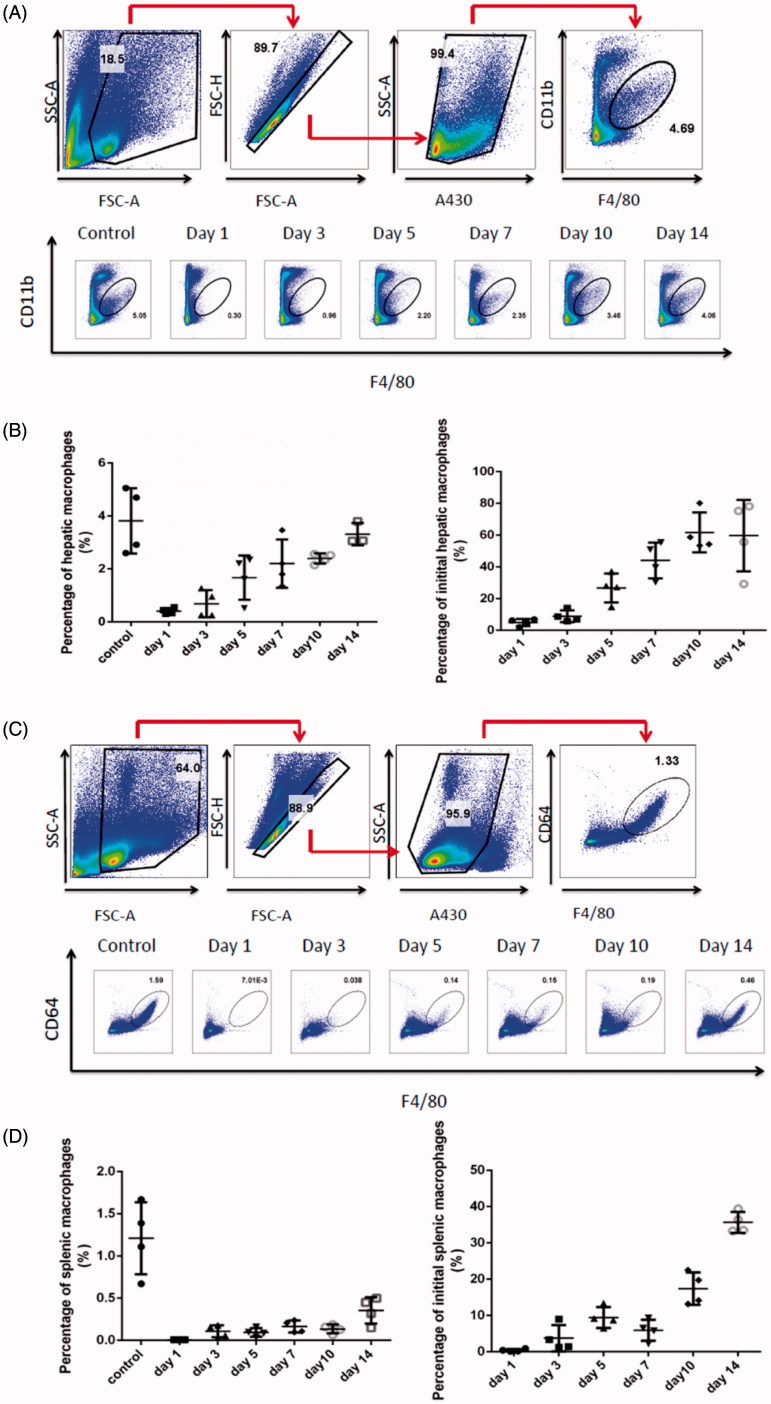
Evaluation of macrophages in the liver after intravenous injection of clodrolip. The flow cytometry analysis strategy of macrophages in the liver (A). The percentage of hepatic macrophages at various times (B). The flow cytometry analysis strategy of macrophages in the spleen (C). The percentage of splenic macrophages at various times (D).

To determine the number of macrophages after parenteral administration of clodrolip, an evaluation of residual macrophages vs. time in liver was carried out. The strategy for flow cytometry analysis of residual macrophages in liver was displayed in Figure 2(A). After careful analysis of macrophages isolated from liver using flow cytometry, we found the percentage of CD11b positive cell population on day 1 reduced from 3.58% to 0.044% in clodrolip group compared with the control group; as also shown in Figure 2(B), in comparison with the number of macrophages in the control group, macrophages in the liver were decreased to a certain degree and the hepatic macrophages only accounted for less than 0.1% when the mice were treated with clodrolip in advance. The results disclosed that intravenous injection of clodrolip can lead to a distinct depletion of hepatic macrophages on day 1. Moreover, the strategy for flow cytometry analysis of splenic macrophages was displayed in Figure 2(C). We found the percentage of CD64 positive cell population on day 1 reduced from 1.28% to 0.003% in clodrolip group compared with the control group; as also shown in Figure 2(D), in comparison with the number of macrophages in the control group, macrophages in the spleen were thoroughly depleted and the percentage of splenic macrophages nearly reached to zero on day 1 when pretreated with clodrolip. Furthermore, we even realized that the depletion of macrophage could at least last for 3 d after single injection of clodrolip in that the ratio of macrophages within 3 d was still below 10% both in spleen and liver. However, macrophages started to repopulate in the liver and the number of them was gradually enhanced and retrieved about 20–40% of the initial population from day 10 to 14. Hence, in order to provide a continuous depletion of macrophages, it had better to administrate clodrolip more than once.

### 3.3. Characterization of PTX-PLGA nanoparticles

PTX, an established potent antineoplastic drug, exhibits better therapeutic capability against many types of carcinomas via stabilization microtubule polymer, disturbance microtubule disassembly, and consequently induces cell apoptosis. However, there are some shortages needed to be addressed such as the poor water solubility of PTX and the potential toxicity induced by non-distribution to the desired sites. Now with the development of nanotechnology, nanoparticles delivery systems have shown superior advantages in improvement of bioavailability of poor soluble drugs and increase targeting over the conventional dosage forms. PLGA-based nanoparticles, as one of the most successful nanocarrier systems, have been widely applied in the field of drug delivery for their particularly biocompatible and biodegradable properties.

**Figure 3.**
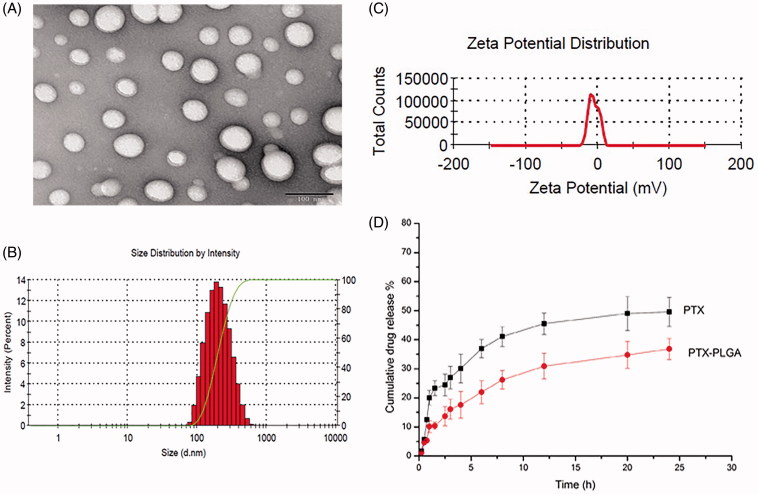
The TEM morphology (A); particle size distribution (B); zeta potential (C); and *in vitro* drug release profiles (D) of PTX-PLGA.

When considering the fact that the active ingredient of PTX and the polymer PLGA could be dissolved simultaneously in organic solvents, we utilized emulsification solvent evaporation technique for the preparation of PTX-PLGA nanoparticles which allowing to encapsulate lipophilic drugs. The TEM results shown in Figure 3(A) disclosed that the prepared PLGA nanoparticles were spherical structure. As depicted in Figure 3(B,C), PTX-PLGA nanoparticles possessed uniformed size distribution with a mean particle size of about 210 nm, the zeta potential was nearly neutral as there were no ionic stabilizer present in the formulation. The DL and EE of the nanoparticles were 7.2% and 81.6% respectively when analyzing the content of PTX by HPLC. As shown in Figure 3(D), the cumulative release of PTX in PTX PLGA nanoparticles exhibited a sustained-release profile compared with free drug. The cumulative release amount of PTX from PLGA nanoparticles reached to less than 40% within 24 h. The sustained drug release profiles could be related with the erosion of polymer material of PLGA.

### 3.4. Pharmacokinetic studies of PTX in mice after depletion of macrophages

**Figure 4.**
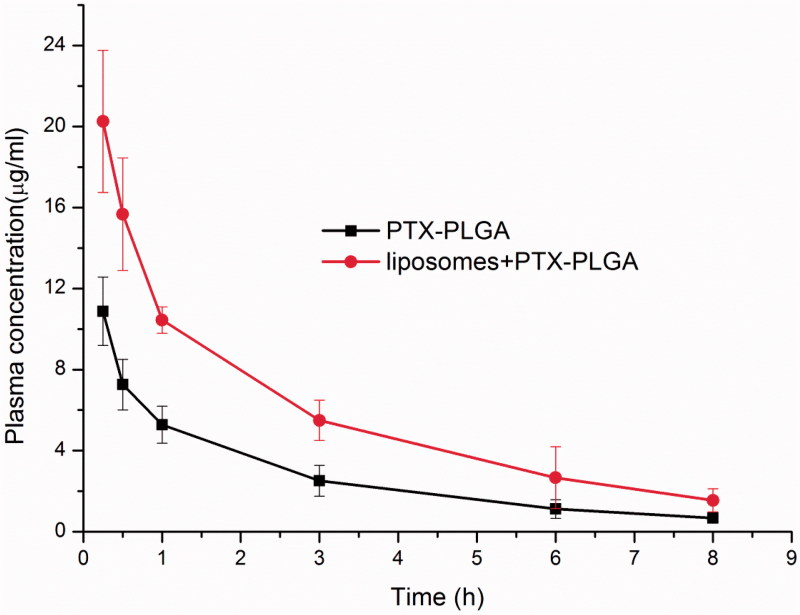
Plasma concentrations versus time profile of PTX in mice with or without depletion of macrophages.

After depletion of macrophages via pre-administration of clodrolip, the plasma concentration of PTX after injection of PTX PLGA nanoparticles (5.0 mg/kg) in mice was measured. Two distinctive curves of the mean plasma concentrations versus time of PTX were shown in Figure 4. From this figure, we found that the plasma concentrations of PTX in macrophages depletion groups were distinctly higher than that of control group without depletion macrophages at all sampling time and the drug concentration of PTX in mice after depletion of macrophages were enhanced nearly twofold when compared with control groups. Pharmacokinetic parameters by non-compartmental analysis were summarized in Table 1. From these results, we observed that there were significantly different parameters present in the macrophages depletion group and the control group. In comparison with the control group, the AUC of PTX in the macrophages depletion group increased 2.11-fold. Moreover, the MRT of PTX in the macrophages depletion group (3.2 h) was much longer than that of control group (2.1 h). The total clearance (*CL*) and *Vd* (apparent volume of distribution) of PTX in the macrophages depletion group were significantly less than those in the control. These pharmacokinetic results gave us a hint that the approach of depletion of macrophages can confer the nanoparticles *in vivo* the long circulation properties and can be used as an effective strategy to improve bioavailability of those administrated by intravenous route.

**Table 1.** Pharmacokinetic parameters of PTX in depletion of macrophages group.

Parameter	Depletion of macrophages	Control group
AUC_0→∞_ (mg/L h)	11.04 ± 3.26*	5.214 ± 1.23
*CL* (mL/h/kg)	90.55*	191.79
*Vd* (mL/kg)	291.09 ± 12.7*	525.95 ± 34.1
MRT (h)	3.21 ± 0.13	2.74 ± 0.18

PTX: paclitaxel; AUC_0→∞_: area under the plasma concentration time curve from zero to infinity; *CL:* clearance; *Vd:* volume of distribution; MRT: mean residence time. Statistical significance compared with reference; *p < .05. Data are represented as the mean ± SD.

### 3.5. Biodistribution studies

**Figure 5.**
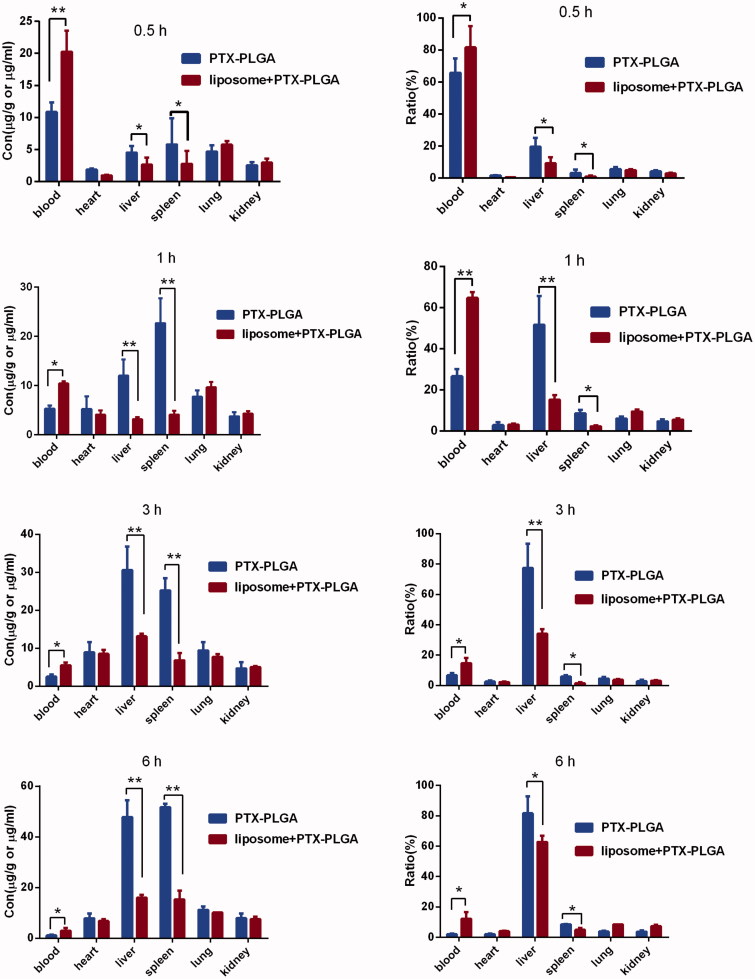
Drug distribution profile of PTX in mice with or without depletion of macrophages at various times.

To evaluate the accumulation of systemic delivery of PTX-PLGA nanoparticles in the MPS organs (such as the liver and spleen), the biodistribution test of PTX in blood and major organs in C57BL/6J mice were probed after administration of 5 mg/kg of PTX-PLGA nanoparticles in mice with or without depletion of macrophages. The biodistribution profiles of PTX were described by parameters such as tissue concentration (μg/g tissue; Figure 5, left column) and the relative tissue dose accumulation ratio (%) (Figure 5, right column). The concentration changes of PTX in the blood showed the similar tendency to the pharmacokinetic experiments, when the macrophages were depleted, the drug concentrations of PTX presented in the blood stream were much higher than those in the control group. In the initial 15 min, the concentration of PTX reached to nearly 80% by macrophages depletion against 62% in the control group. While within 1 h period, a rapid decline of drug concentration was observed in the group without macrophages depletion; this suggested that the exogenous nanoparticles would initiate the immunological protective mechanism of the body and then were sequestered by the macrophage phagocytic system (MPS) within short durations after intravenous administration.

### 3.6. Induction of significant antitumor efficacy in B16/F10 melanoma models

**Figure 6.**
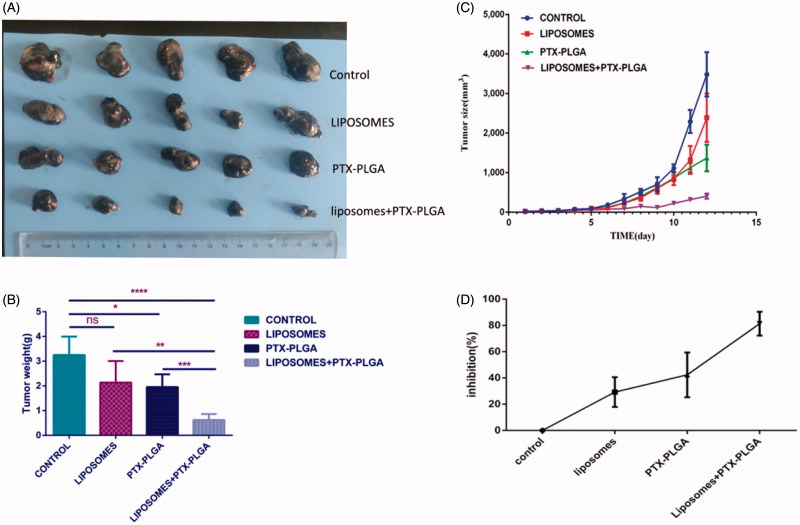
*In vivo* antitumor performance of PTX-PLGA in B16 xenografted C57 mice with or without depletion of macrophages. Representative tumor tissues after treatment (A); average tumor weight at the experimental endpoint (B); tumor size over the treatment regimen (C); relative inhibition ratio of tumor growth (D).

To validate whether depletion of macrophages can be used as a synergetic therapeutic approach to inhibit tumor growth, when PTX-PLGA nanoparticles were injected intravenously each twice day for four times, therapeutic efficacy of this regimen was evaluated by monitoring tumor growth in the mice bearing B16/F10 tumors with or without macrophages depletion. The efficacy of parenteral administration of clodrolip and/or PTX-PLGA nanoparticles was found to obviously inhibit tumor growth, as illustrated in Figure 6(A–C). Actually, in comparison with PBS-treated control group, the tumor volumes in mice either pretreated or not treatment with clodrolip were obviously decreased after administration of PTX-PLGA nanoparticles. Furthermore, the group treated with combinatorial of parenteral administration of clodrolip and PTX-PLGA nanoparticles showed superior antitumor activity. As distinctly evident in Figure 6(D), after depletion of macrophages and subsequent administration of PTX-PLGA nanoparticles showed considerable inhibition rate of tumor growth, the inhibition ratio was nearly reached to 80%; while the inhibition rate generated by sole administration with PTX-PLGA nanoparticles only accounted for about 40%. However, most interesting phenomenon is that the administration of clodrolip can also display inhibition of tumor growth.

### 3.7. Adverse effects of the combination regimen of macrophage depletion and PTX-PLGA treatment

**Figure 7.**
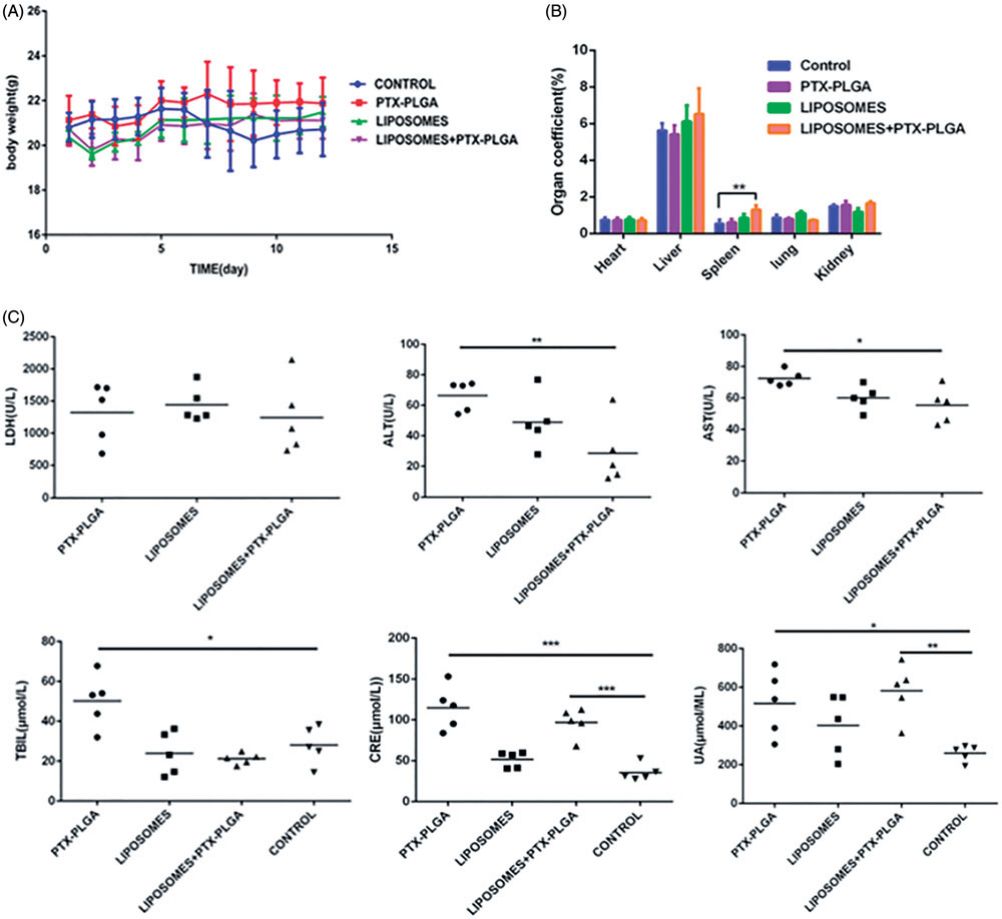
Evaluation of drug-related toxicity. Change in body weight over the regimen (A); organ coefficients (B); hepatotoxicity analysis of serum markers such as lactic dehydrogenase (LDH), aspartate transaminase (AST), alanine transaminase (ALT), total bilirubin (TBIL), uric acid (UA), and creatinine levels (CRE) in mice (C).

In this experiment, we evaluated the changes of the body weight, organ coefficients, and biochemical alterations for determination the side effects of the combination regimen. The body weight curve in Figure 7(A) showed that there was no manifest and severe body weight loss observed in the experimental groups except for the control group. As a significant body weight loss was the typical symptom associated with the side effects of chemotherapy during tumor progressive period. The result exhibited that incorporation model drug PTX into the PLGA nanoparticles can diminish the toxicity of the free PTX; also the formed core-shell structure of PLGA nanoparticles can control drug release from the matrix of the nanoparticles and decrease the toxicity.

In addition, the major organs were harvested at the end of experiments and precisely weighed to calculate the organ coefficient. Organ coefficient is considered as another most sensitive index for estimation drug toxicity, from Figure 7(B) we found that there were slightly change in the major organs such as heart and liver; while we noticed that the organ coefficient of spleen increased and showed significantly statistical difference. We did not yet understand the mechanisms and just assumed that the alterative organ efficient of spleen was involved in removing the macrophage presented in the spleen.

**Figure 8.**
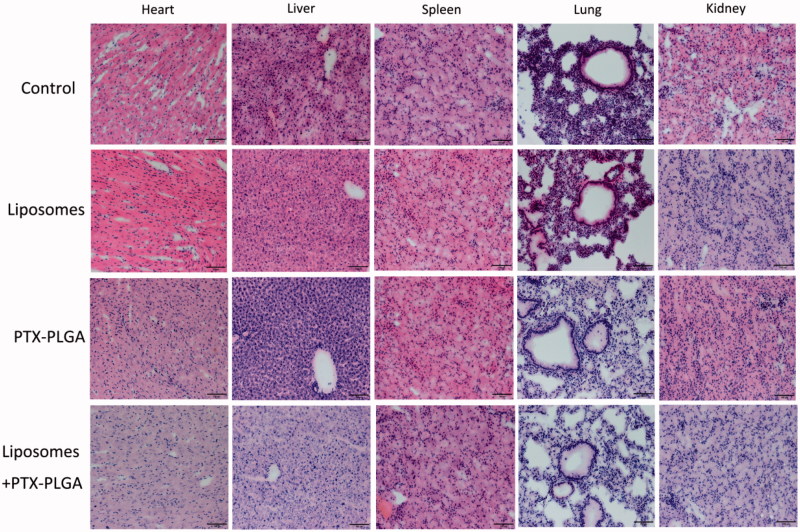
Histopathologic examination of major organs after treatment (20×).

Moreover, we also performed a toxicology assessment using hematological biochemistry assays on experimental animals. Some biochemical markers such as AST, ALT, TBIL, UA, and CRE were elevated as an apparent indicator for liver or kidney injury (Figure 7(C)). Radi et al. (2011) reported increased serum enzyme levels were not a symbol of hepatic or skeletal muscle injury in that kupffer cells exerted an important role in the clearance of several serum enzymes. Therefore, the increase in the serum enzymes levels may be related with the result of depletion of macrophages instead of hepatic injury. Histological sections of tissue were showed in Figure 8; the results disclosed that no morphological changes in major tissues were detected in HE stained sections by microscopic examination, indicating that the vital organs were not seriously damaged in this treatment approach.

## 4. Discussion

Nanoparticles have already been fueled more attention and broadly utilized as effective vehicles for delivery active pharmaceutical ingredients to specific targeted regions, such as tumor sites. Application of PLGA to fabricate nanoparticles is considered as a promising tool in the field of cancer therapy because of these polymers with better biodegradability and biocompatibility, DL capability, controlled drug release properties. Traffic of systemically administered nanoparticles in the blood stream will still encounter many physiological barriers and hence the potential of delivered nanoparticles does not function properly. Now, liver is regarded as the primary barrier for systemically administrated nanoparticles, as up to 90% of the dose is usually sequestrated by this organ (Samuelsson et al., 2017). The reason why enable nanoparticles accumulate in the liver may be attributed to the innate phagocytes and the large amounts of vascular networks presented in the liver. As a group of professional phagocytic cells, in particular kupffer cells, the primary macrophages resident in the liver, are considered to be responsible for capture of foreign nanoparticles (Gustafson et al., 2015). Therefore, the unavoidable and prompt capture of nanoparticles by kupffer cells results in ‘off-targeted clearance’ and subsequently cannot dispense more drugs into the desired sites. When the nanoparticles arrive at the liver and filter through the hepatic sinusoids, as a natural safeguard, the kupffer cells may inevitably capture nanoparticles and impede them distributing to other organs. Therefore, intended to delay nanoparticles clearance, modulation of professional phagocytes of the MPS resident in the liver as an effective therapeutic strategy to improve anti-tumor efficacy was focused on.

In order to weaken or overcome the innate sequestration effect presented in the MPS, the common once used process named PEGylation, which conjugating the hydrophilic polyethylene glycol (PEG) chain to the surface of nanoparticles, was mainly introduced as a preferential approach for evasion MPS clearance. While more and more evidences disclosed that this strategy was not favorable for systemic drug delivery in that PEGylation of nanoparticles would activate the complement system and lead to the formation of PEG-specific antibodies, in which accelerated nanoparticle clearance from the circulation (Knop et al., 2010; Moghimi et al., 2010). Additionally, the long hydrophilic PEG chain might diminish interactions with all types of cells, without any exception including combination with cancer cells (Hatakeyama et al., 2013). In a word, there is a requirement for probe another alternative strategy to ameliorate drug delivery.

In this study, we demonstrated that the use of clodrolip could effectively deplete kupffer cells within 1 day; in addition we noticed that this means of macrophages depletion was in a reversible manner as the population of macrophages repopulated to 40% in comparison with the control group on day 14. This result gave us a hint that this strategy did not bring about permanent impairment for MPS and just shut off the phagocytic function of macrophages for a short duration.

Based on the fact that clodrolip can effectively harness the function of macrophages in the liver, we next evaluated whether this pretreatment approach of clodrolip could better the bioavailability of systemically administrated nanoparticles. Date in Figure 4 showed that the concentration of PTX in blood was almost elevated to 2.3-fold in the macrophages depletion groups by clodrolip within 1 h. The total concentration of PTX *in vivo* included the parts resident in the vessel and those entered into the organ over a period of time. If we suppressed the function of innate macrophages that compete for capture the nanoparticles, which in theory allow a higher concentration available in the blood. Therefore, this outcome demonstrated that the enhanced PTX concentration should be involved in the impaired activity of kupffer cells in the liver to inhibit uptake of PTX-PLGA nanoparticles. Also as shown in Figure 5, upon intravenous injection, PTX-PLGA nanoparticles mainly accumulated in MPS organs such as liver and spleen due to the process of opsonization. Just as anticipated, the pharmacokinetics parameters and organ distributions of PTX in the macrophage depletion group were significantly changed. Importantly, compared to the control groups, the concentrations of PTX distributed in liver significantly decreased from nearly 55% to 18% within 1 h and from 80% to 36% within 3 h, respectively. Moreover, at all the sample collecting time points, the administrated drugs were mainly concentrated in the liver and spleen. When active ingredients-loaded nanoparticles circulated in the blood vessel and passed through these organs, the prompt capture took place resulted from the MPS and consequently would bring about outstanding accumulation in these locations, especially in the liver, where a larger amount of resident macrophages present in. On the contrary, PTX contents distributed in the liver and spleen were significantly decreased if the macrophages had been cleared. In summary, we believe that the phenomena of boosted concentration and the decreased drug distribution in the liver should be related with dysfunction of macrophages.

In the melanoma-bearing mice model, antitumor effect of this treatment strategy using the combination of clodrolip with PTX-PLGA showed a remarkable reduction of tumor growth with respect to controls. As shown in Figure 6, we realized that macrophage depletion approach presented an encouraging therapeutic performance *in vivo*, which suggesting that direct removal of macrophages in the liver is a promising strategy for cancer therapy. As only maintenance enough plasma concentrations can allow more and more drugs to be permeated into the tumor via the EPR effect and high drug concentrations are available to erase tumor. As kupffer cells were depleted, the increased plasma concentrations of PTX provided effective high concentration in the tumors for long period and subsequently lead to significant growth inhibition effect. Also from another opinion, tumor-associated macrophages (TAMs) are related to tumor progression and invasion; some researchers have already demonstrated that clodrolip was sufficient to in deplete macrophages in different disease models (Bacci et al., 2009; Zhan et al., 2014). Furthermore, a novel clodronate-containing liposomal formulation was developed to deplete TAMs in primary and metastatic murine melanoma models and resulted in better antitumor efficacy (Piaggio et al., 2016). We also found that the experimental group with mere injection of clodrolip has the sign to inhibit tumor growth; although the tumor inhibition did not show significant difference in comparison with control group.

As we all known that macrophages are integral and vital components for composition of the innate immune system. The data that macrophage depletion increased the delivery efficacy to the tumor are promising but one concern may be questioned whether macrophage depletion would cause systemic toxicity? Although pretreatment of clodrolip to suppress macrophages represented a double-edged approach, the thorough evaluation of adverse effect on depletion of kupffer cells demonstrated this strategy did not lead to evident tissue injury.

## 5. Conclusions

We have developed a macrophage-pretreatment approach for improvement nanoparticles tumor delivery. Clodrolip could effectively deplete macrophages presented in the liver; those cells were reported to sequestrate the largest amount of circulating nanoparticles. We discovered that the removal of the liver macrophages would bring out a superior efficiency for better the biodistribution and performance of nanoparticle delivery systems. Also, treatment of macrophage preconditioning of mice bearing melanoma with the combination of PTX-PLGA nanoparticles resulted in an encouraging antitumor efficacy in comparison with respect to other groups. It is worth mentioned that the combinatorial regimens of clodrolip and PTX-PLGA chemotherapy have demonstrated acceptable toxicity in incidence of adverse effects. In conclusion, the encouraging results from this study inspire the generation of a rational strategy to focus on microenvironmental priming for modulation of innate immunity and to improve delivery efficiency of nanoparticles.
